# Comprehensive Statistical Evaluation of Etest^®^, UMIC^®^, MicroScan and Disc Diffusion versus Standard Broth Microdilution: Workflow for an Accurate Detection of Colistin-Resistant and *Mcr*-Positive *E. coli*

**DOI:** 10.3390/antibiotics9120861

**Published:** 2020-12-03

**Authors:** Isidro García-Meniño, Pilar Lumbreras, Pablo Valledor, Dafne Díaz-Jiménez, Luz Lestón, Javier Fernández, Azucena Mora

**Affiliations:** 1 Laboratorio de Referencia de Escherichia coli (LREC), Departamento de Microbioloxía e Parasitoloxía, Facultade de Veterinaria, Universidade de Santiago de Compostela (USC), 27002 Lugo, Spain; isidro.garcia@usc.es (I.G.-M.); dafne.diaz@usc.es (D.D.-J.); luz.cambeiro@usc.es (L.L.); 2 Instituto de Investigación Sanitaria de Santiago de Compostela (IDIS), 15706 Santiago, Spain; 3 Servicio de Microbiología, Hospital Universitario Central de Asturias, 33011 Oviedo, Spain; UO231178@uniovi.es; 4 Grupo de Microbiología Traslacional, Instituto de Investigación Sanitaria del Principado de Asturias (ISPA), 33011 Oviedo, Spain; 5 Research & Innovation, Artificial Intelligence and Statistical Department, iAST™, 33007 Oviedo, Spain; pablo.valledor@iast.ai

**Keywords:** *E. coli*, *mcr*, colistin, ST131, AST, MicroScan, BMD

## Abstract

Four colistin susceptibility testing methods were compared with the standard broth microdilution (BMD) in a collection of 75 colistin-susceptible and 75 *mcr*-positive *E. coli*, including ST131 isolates. Taking BMD as reference, all methods showed similar categorical agreement rates (CA) of circa 90%, and a low number of very major errors (VME) (0% for the MicroScan system and Etest^®^, 0.7% for UMIC^®^), except for the disc diffusion assay (breakpoint ≤ 11 mm), which yielded false-susceptible results for 8% of isolates. Of note is the number of *mcr*-positive isolates (17.3%) categorized as susceptible (≤2 mg/L) by the BMD method, but as resistant by the MicroScan system. ST131 *mcr*-positive *E. coli* were identified as colistin-resistant by all MIC-based methods. Our results show that applying the current clinical cut-off (>2 mg/L), many *mcr*-positive *E. coli* remain undetected, while applying a threshold of >1 mg/L the sensitivity of detection increases significantly without loss of specificity. We propose two possible workflows, both starting with the MicroScan system, since it is automated and, importantly, it categorized all *mcr*-positive isolates as colistin-resistant. MicroScan should be followed by either BMD or MIC-based commercial methods for colistin resistance detection; or, alternatively, MicroScan, followed by PCR for the *mcr* screening.

## 1. Introduction

Colistin is one of the so-called last line antimicrobials. Due to the continuous emergence of multidrug resistance among Gram-negative bacteria, the scientific community has renewed interest in polymyxins as a therapeutic option, despite its toxicity, exemplified by the significant increase of it being prescribed in human medicine in the recent years [1,2]. Resistance to polymyxins has been reported in bacterial isolates recovered from humans, animals, food, and the environment [2,3]. Farm animals, poultry, and swine in particular, have been identified as the main reservoir of resistance to these drugs worldwide [2,4,5,6]. Colistin resistance primarily occurs through chromosomal mutations (in the genes encoding two-component systems PmrAB and PhoPQ, or in the MgrB regulator), and/or by plasmid-borne mobile colistin resistance genes (*mcr*), which have shown a high capacity for dissemination [2]. To date, ten *mcr* genes (*mcr-1* to *mcr-10*) have been described, mainly in Enterobacteriaceae species [7]. Furthermore, colistin resistance has been strongly linked to the globally mobilized gene *mcr-1,* presumably due to the over-use of polymyxin in human and veterinary medicine [8]. By contrast, *mcr-4* is reported to be widespread in swine and poultry populations, and, in several cases, *mcr-4* is found along with *mcr-1*, *mcr-3*, or *mcr-5* [9]. Similar to the situation in other countries, a high prevalence of *mcr-1* and *mcr-4* within *E. coli* isolated from food-producing animals, especially in poultry and pig farming, with *mcr-4.5* and *mcr-4.2* as the most frequent *mcr-4* variants, has been described in Spain [5,6]. García-Meniño et al., (2018) [6] also reported, for the first time, *mcr-1 E. coli* isolates recovered in pigs belonging to the pandemic lineage ST131.

Due to the difficulties with the performance, reproducibility, and precision of the currently available methods for colistin susceptibility testing [9], evaluation and comparison of these methods on wide collections can help to define more practical algorithms. It is also critical to avoid inaccuracies, especially very major errors (VME), which could lead to therapeutic failures. Currently, both the Clinical and Laboratory Standards Institute (CLSI) and European Committee on Antimicrobial Susceptibility Testing (EUCAST) advise against the use of the disc diffusion method for polymyxin susceptibility testing, and identify the broth microdilution method (BMD) as the only recommended method for this purpose (https://eucast.org/). Other studies have evaluated the efficiency of different susceptibility-testing methodologies for the detection of colistin-resistant isolates, however, most of them were performed on a limited number of isolates and lacked variety of *mcr* types [10,11,12,13]. In the present study, we analyzed different colistin susceptibility testing methods on a large collection of colistin-susceptible and *mcr*-positive *E. coli* of types 1, 2, 4, and 5, including ST131 *mcr*-positive and negative isolates. The aim was to perform a comprehensive statistical evaluation of four colistin susceptibility testing methods versus the standard BMD, in order to provide a suitable workflow for the accurate detection of colistin-resistant and *mcr*-positive *E. coli*.

## 2. Results

**Figure 1:**
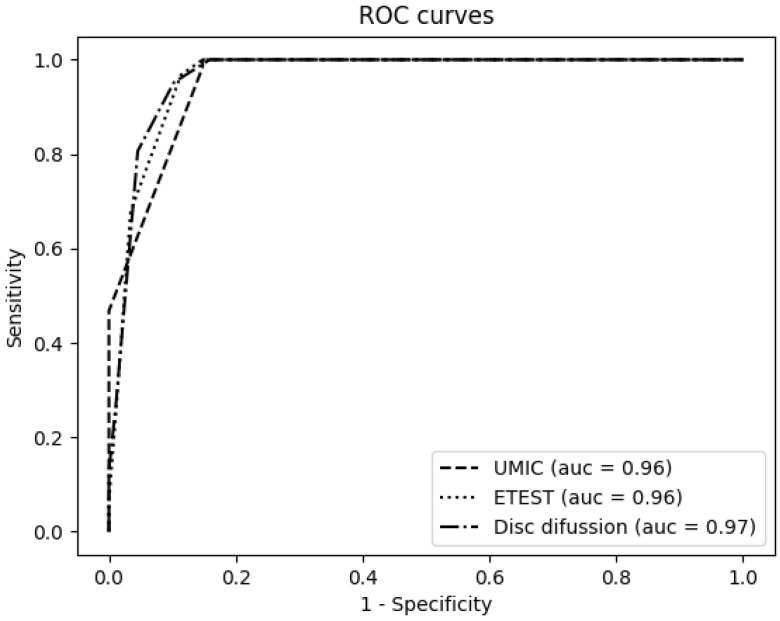
Receiver operating characteristic (ROC) curves for the Etest^®^, UMIC^®^ and disc diffusion using the broth microdilution method (BMD) as reference.

Tables S1 and S2 show the colistin susceptibility results for each isolate and method. The performance of each testing method was represented by receiver operating characteristic (ROC) curves, as shown in Figure 1, except for the MicroScan system, for which the number of dilutions assayed is not enough to build these curves. As a result, the UMIC^®^, Etest^®^ and disc diffusion displayed good area under curve (AUC) values (0.96 for UMIC^®^ and Etest^®^, 0.97 for disc diffussion), without significant differences between them.

**Figure 2:**
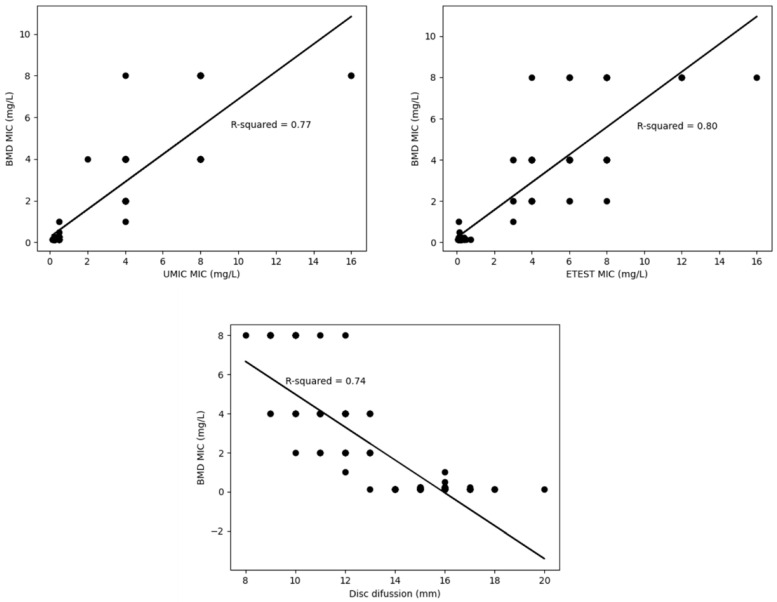
Correlation of each testing method versus the reference (BMD) represented by regression curves and R-squared.

However, the results obtained in the lineal regression analysis were varied and did not show good correlation with the reference method (BMD) (Figure 2), being the R-squared values low, especially for the disc diffusion method (0.74).

**Figure 3:**
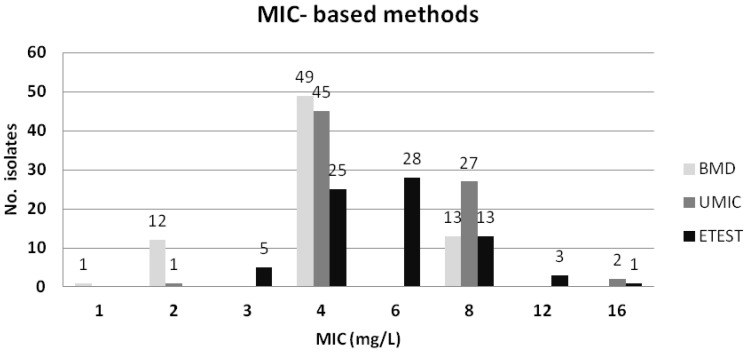
Colistin MICs for the 75 *mcr*-positive *E. coli* evaluated with BMD, UMIC^®^ and Etest^®^.

The essential agreement (EA), categorical agreement (CA), major error (ME), and very major error (VME) for all tests, in comparison with the standard BMD, are summarized in Table 1. Taking BMD as reference, all methods showed similar CA of circa 90%, and a low number of VME (0% for the MicroScan system and Etest^®^, and 0.7% for UMIC^®^), except for the disc diffusion assay with the previously recommended breakpoint of ≤11 mm [14], which yielded false-susceptible results for 12 (8%) isolates (Table S1). All methods exceeded the ME rates recommended by CLSI (≤3%) [15], with the exception of the disc diffusion (using the breakpoint of ≤11 mm). However, when applying a breakpoint of ≤13 mm for the disc diffusion method, the CA and the VME values were the same as for the MicroScan system (90.7% and 0%, respectively), although the ME was higher than recommended. The MIC values for *mcr*-positive isolates obtained by the BMD, UMIC^®^ and Etest^®^ methods are represented in Figure 3.

It is of note that the standard BMD categorized as susceptible 13 (17.3%) of the 75 *mcr*-positive isolates according to the EUCAST 2020 [16] and CLSI [17] breakpoints (resistant >2 mg/L), although 12 of those 13 showed a MIC value = 2 mg/L. In contrast, the MicroScan system and Etest^®^ identified all 75 isolates as colistin-resistant, as displayed in Table 1 and Table S1. On the other hand, the standard BMD, and the compared methods, identified as susceptible the 75 *mcr*-negative control isolates, with the exception of the MicroScan system which displayed a value of 4 mg/L for a single isolate (Table S2). Importantly, all 11 ST131 *mcr*-1-positive and the 24 ST131 *mcr*-negative *E. coli* were identified correctly as colistin-resistant and susceptible, respectively, by the reference BMD method and the other broth dilution methods. A single ST131 *mcr-1* isolate was identified as susceptible by the disc diffusion method.

**Figure 4:**
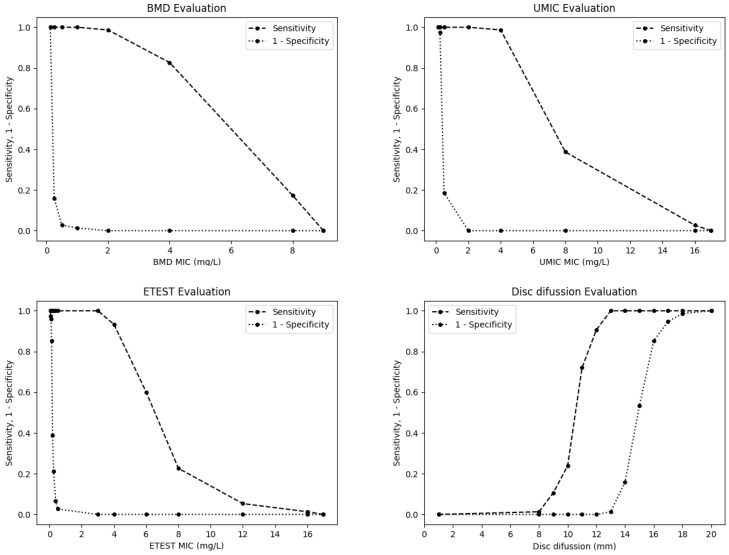
Sensitivity and specificity of the BMD, UMIC^®^, Etest^®^ and disc diffusion methods for the detection of *mcr*-carrying *E. coli* isolates at different thresholds.

The sensitivity and specificity of the evaluated methods for detection of *mcr*-carrying *E. coli* at different thresholds are shown in Figure 4. Applying a cut-off value of 2 mg/L (which is equivalent to point ≥4 in the graphic representation), the sensitivity and specificity of BMD are 82.67% and 100%, respectively; while, using a threshold of 1 mg/L (point ≥2 in the graphic representation), the sensitivity increases until 98.7% and maintains 100% of specificity. For the disc diffusion method, when applying the Gales et al. [14] cut-off value of resistant ≤11 mm, the sensitivity and specificity achieved are of 72% and 100%, respectively; while, using the cut-off value proposed here of resistant ≤13 mm, the sensitivity increases until 100% with a specificity of 98.7%.

## 3. Discussion

Despite the increasing use of colistin in human medicine due to the emergence of MDR Gram negative bacteria, susceptibility testing for this antimicrobial is still challenging. Suitable protocols are still needed for the accurate detection of resistant isolates in clinical laboratories. CLSI and EUCAST recommend the BMD as the gold standard for colistin susceptibility testing. Nevertheless, this method is not automated and is very time-consuming, not being available in most hospitals.

As expected, the colistin disc diffusion testing applying the Gales et al. [14] cut-off value of ≤11 mm, showed a poor correlation with BMD (CA of 89.3%) and an unacceptable number of false susceptible isolates, resulting in a significantly high rate of VME (8%). This fact could lead to therapeutic failures, due to the inaccurate administration of this drug. It has been previously reported that agar-based methods perform poorly, being unreliable to measure colistin susceptibility because of its cationic nature and unpredictable diffusion through *agar* [11]. However, applying the here proposed cut-off value of 13 mm, the VME and ME rates would be 0% and 9.3%, respectively. The results achieved with the broth dilution methods, MicroScan and UMIC^®^, were significantly more accurate than those obtained with the disc diffusion assay, showing a good correlation with BMD, and with fewer VME (0% for MicroScan and 0.7% for UMIC^®^). Interestingly, very similar CA and VME rates were observed with the Etest^®^ (91.3% and 0%, respectively).

Currently, neither CLSI nor EUCAST recommend the disc-diffusion method and therefore, there is no reference of zone diameter breakpoints. However, this method has been used as a screening test for years, and it is still widely performed in many laboratories. Regarding gradient diffusion methods, their efficacy is controversial for colistin susceptibility testing, although some studies have validated and recommended them as accurate alternative tests, demonstrating good concordances with the reference method [18,19]. In contrast, other authors have highlighted inaccuracies in the gradient diffusion methods, including high rates of VME: 53.6% [10] and 12.0% [20]. Furthermore, depending on the study, it has been claimed that the Etest^®^ overestimates or underestimates MICs [11,19]. In the present study, we did not detect any VME for the Etest^®^, and the most outstanding fact for both Etest^®^ and MicroScan is that they were able to detect all *mcr*-positive isolates. In contrast, the BMD method did not identify resistance in 17.3% of the isolates. In agreement with our observation, other authors also reported favorably on the use of MicroScan or other automated systems, such as the BD Phoenix for the screening of *mcr*-positive *E. coli* isolates [10,13,19,21], which are currently of high concern. Furthermore, different commercial and user-friendly broth dilution based tests have been assessed (comASp^®^, colistin MAc test^®^, Sensititre, Sensitest, MICRONAUT, UMIC^®^) with promising and reproducible results that correlated well with BMD [11,13].

Regardless of the parameters discussed above, it is important to bear in mind that the regression curves built for the Etest^®^, UMIC^®^ and the disc diffusion showed low R-squared values, which means that the exact MIC obtained by the reference method cannot be extrapolated from the results obtained by any of these methods, since data are varied with no linear relationship between them.

It is outstanding, as mentioned above, that 13 *mcr*-carrying isolates were assigned as susceptible by the BMD reference method, although 12 of them showed MIC values close to the breakpoint (2 mg/L). Most studies have reported MIC values to polymyxins of ≥2 mg/L for isolates carrying *mcr* genes [10,11,12,13,21]. However, the presence of *mcr* genes does not mean phenotypical expression of colistin resistance or high MIC levels. In fact, *mcr-1*-positive *E. coli* isolates usually display a low-level of colistin resistance (2–8 mg/L) [2,3,22]. As an example, 53 (66.3%) of the 80 *mcr-1* isolates which were collected from fecal samples of turkeys, chickens, pigs, and cattle in Poland exhibited a colistin MIC of 2 mg/L [23]. Similarly, (17/26; 65.4%) *mcr-1*-positive *E. coli* isolates of human clinical origin in Brazil showed a polymyxin B MIC of 2 mg/L [24]. It is worth highlighting that the 11 *mcr-1*-positive *E. coli* of the pandemic clone ST131 of our study, were identified as colistin-resistant by the reference method BMD, and all but one also tested as resistant by the disc diffusion method. The acquisition of *mcr-1* by this specific clone means a summative risk with respect to treatment failure due to its special feature of congregating virulence, resistance traits, and its high dissemination capability. Since ST131 is implicated in many community- and hospital-acquired urinary tract infections (UTIs) [25], the accurate identification of colistin-resistance in this high-risk lineage is therefore critical.

Low levels of colistin resistance, or even no phenotypic expression, have been observed for other *mcr* genes, such as *mcr-3* and *mcr-4* [26]. Although the clinical implications for the polymyxin therapy against the *mcr*-positive isolates with low MICs remain unclear, it is important to take into account that the *mcr-1* gene can facilitate the selection of chromosomal mutations, which eventually leads to high-level of colistin resistance [22]. We underline here that, using the current cut-off values (>2 mg/L), many of the *mcr* positive isolates that might contribute to the silent dissemination of these genes would remain undetected. On the contrary, if a cut-off value of >1 mg/L is applied, 98.7% of *mcr*-positive isolates are detected without loss of specificity.

We did not observe differences in MIC values between the 25 *mcr*-positive genomes with no known chromosomal mutations, and the 16 that had a summative presence of *mcr* genes and chromosomal mutations in the *pmr*A (*pmr*A S39I, 2 isolates) or *pmr*B (*pmr*B V161G, 14 isolates) (Table S1). Similarly, a previous study did not found any summative effect in one *mcr*-1 positive *E. coli* isolate and carrier of a chromosomal mutation *pmr*B V161G (MIC = 2 mg/L) [23].

We are conscious that this study has the limitation of being mostly descriptive. Further investigation is needed on the internal molecular mechanisms of *mcr*-positive isolates to fully understand their phenotype expression of colistin resistance. However, we have to point out in comparison to previous studies, the high number of *mcr*-positive and negative isolates analyzed here (75 each), including a wide variety of *mcr* genes (*mcr-1*, *mcr-2*, *mcr-4*, and *mcr-5*), as well as the comprehensive statistical analysis performed.

## 4. Materials and Methods

A set of 150 *E. coli* was chosen from a collection of isolates recovered from food (meat), food-producing animals (pigs, chicken, and cattle), and wildlife in the period 2006–2020 [4,5,6,27,28]. The selection included 75 colistin-susceptible and 75 *mcr*-positive isolates carrying different *mcr* types: *mcr*-1 (43 isolates), *mcr*-2 (1 isolate), *mcr*-4 (*mcr*-4.1, 1 isolate; *mcr*-4.2, 17 isolates; and *mcr*-4.5, 6 isolates), *mcr*-5 (2 isolates), *mcr*-1/*mcr*-4 (3 isolates), and *mcr*-4/*mcr*-5 (2 isolates) (Table S1).

The accuracy of antimicrobial susceptibility tests (ASTs) was measured using categorical agreement (CA) and essential agreement (EA). CA is the total number of isolates tested using the ASTs that yields a MIC result, and the same categorical interpretation as the BMD MIC result (used as reference). CA discrepancies were subdivided into two types of errors: major error (ME), defined as those isolates found to be resistant using one of the alternative methods but categorized as susceptible by the reference BMD method; and very major error (VME), defined as those isolates found to be susceptible using one of the alternative methods but categorized as resistant by the BMD method. EA is defined as obtaining a MIC value with the evaluated AST that is within 1 log_2_ dilution of the reference BMD MIC value, and it is applicable only to methods that determine MIC values [29]. In the present study, the CA, EA, ME, and VME of four different methods (disc diffusion, Etest^®^, bioMérieux, Marcy l’Etoile, France; UMIC^®^, Biocentric, Bandol, France; and MicroScan WalkAway System, Beckman coulter, Inc., Brea, CA, USA) were calculated for colistin susceptibility testing in comparison with the gold standard BMD method. The diffusion and MIC-based methods (the manual Etest^®^ and UMIC^®^; and the automated MicroScan system) were performed according to the manufacturer’s recommendations, all on the same day, and from the same bacterial inoculums adjusted to the standard 0.5 McFarland. BMD was performed according to ISO standard 20776-1. In brief, 96-well polystyrene plates were filled with cation-adjusted Mueller-Hinton broth (Sigma-Aldrich^®^, Merck, Germany) with colistin concentrations adjusted (Sigma-Aldrich^®^, Merck, Germany) and ranging from 0.125 to 128 mg/L, and then inoculated with a bacterial concentration of ~10^6^ CFU/mL. The results obtained were read and interpreted after 18–24 h of incubation, according to the colistin clinical cut-off values established by EUCAST [16], which considers MICs >2 mg/L as resistant. Following the recommendations of EUCAST, a drug-susceptible *E. coli* strain (ATCC 25922) was included as a quality-control for all the assays (MIC values for colistin = 0.5–1). The disc diffusion susceptibility testing was performed using colistin discs of 10 µg (BIO-RAD). The zone inhibition diameters were interpreted according to Gales et al. [14] (resistant ≤ 11 mm), and according to the new criteria proposed in this paper (resistant ≤ 13 mm), since there is not a standardized cut-off value for colistin in either the EUCAST or the CLSI guidelines.

In order to perform a comprehensive statistical evaluation, receiver operating characteristic (ROC) curves were built to visualize the performance of each testing method, showing the diagnostic ability to identify colistin resistance and their discrimination thresholds. BMD results were used as the sample’s result reference. True positive rate (sensitivity) versus false positive rate (1-specificity) was plotted in the ROC curves, together with the area under the curve (AUC) for each testing method. AUC represents a measure of separability, which shows how much a testing method is capable of distinguishing between classes (colistin-resistant versus colistin-susceptible), and the best method is the one with the highest AUC. Additionally, in order to identify the optimal cut-off value of each testing method to discriminate *mcr*-carrying isolates, a figure showing the evolution of sensitivity and 1-specificity in relation to the threshold changes was plot for each testing method. Finally, regression curves were built to measure the correlation of each testing method versus the reference (BMD) using R-squared (coefficient of determination) and showing the variability in the samples. All these statistical tests were performed using Python programming language (Python Software Foundation. Python Language Reference, version 3.7. Available at http://www.python.org), scikit-learn library and visualize using matplotlib [30].

## 5. Conclusions

Our results denote that applying the current clinical cut-off (>2 mg/L), many *mcr*-positive *E. coli* remain undetected, while applying a threshold of >1mg/L, the sensitivity of detection increases significantly, without loss of specificity. For this reason, we propose two possible workflows, both starting with the MicroScan system, since it is automated, available in numerous clinical microbiology laboratories, and, importantly, it categorized all 75 *mcr*-positive as colistin-resistant. MicroScan should be followed by either BMD or broth dilution commercial methods for colistin resistance detection; or, alternatively, MicroScan, followed by PCR for the *mcr* screening.

## Figures and Tables

**Table 1:** Results of essential agreement (EA), categorical agreement (CA), major error (ME), very major errors (VMEs), sensitivity and specificity for all tests in comparison with the standard BMD.

Methods (Cut-OFF Values)	No. of Isolates (%) Categorized as Resistant	No. of Isolates (%; CI) Exhibiting EA	No. of Isolates (%; CI) Exhibiting CA	No. of Isolates (%; CI) Exhibiting ME	No. of Isolates (%; CI) Exhibiting VME	Sensitivity (%) N = 62	Specificity (%) N = 88
disc diffusion (≤11 mm)	54 (36)	NA	134 (89.3; 84.4–94.2)	4 (2.7; 0.1–5.3)	12 (8; 3.7–12.3)	50 (80.9)	84 (95.4)
disc-diffusion (≤13 mm)	76 (50.7)	NA	136 (90.7; 86.1–95.3)	14 (9.3; 4.7–13.9)	0 (0; 0)	62 (100)	74 (84.1)
UMIC^®^ (>2 mg/L)	74 (49.3)	49 (32.7; 25.2–40.2)	136 (90.7; 86.1–95.3)	13 (8.7; 4.2–13.2)	1 (0.7; 0–2)	61 (98.4)	75 (85.2)
Etest^®^ (>2 mg/L)	70 (46.7)	55 (36.7; 29–44.4)	137 (91.3; 86.6–95.8)	13 (8.7; 4.2–13.2)	0 (0; 0)	62 (100)	75 (85.2)
MicroScan (>2 mg/L)	76 (50.7)	NA	136 (90.7; 86.1–95.3)	14 (9.3; 4.7–13.9)	0 (0; 0)	62 (100)	74 (84.1)

CI, confidence interval; EA, essential agreement; CA, categorical agreement; ME, major error; VME, very major error; NA, not applicable, EA is not applicable to the disc diffusion method or to the MicroScan system (for which the highest dilution assayed is 4 mg/L). Sensitivity and specificity are defined as the ability of detecting resistant and susceptible isolates, respectively.

## Supplementary Materials

The following are available online at https://www.mdpi.com/2079-6382/9/12/861/s1, Table S1: Results of colistin susceptibility for 75 *mcr*-positive *E. coli* isolates using BMD, UMIC, MicroScan, gradient diffusion strip (Etest^®^) and disc diffusion. Table S2: Results of colistin susceptibility for 75 *mcr*-negative *E. coli* isolates using BMD, UMIC, MicroScan, gradient diffusion strip (Etest^®^) and disc diffusion.
