# Supplementary material for: Comprehensive Statistical Evaluation of Etest®, UMIC®, MicroScan and Disc Diffusion versus Standard Broth Microdilution: Workflow for an Accurate Detection of Colistin-Resistant and Mcr-Positive E. coli
Source: Antibiotics (Basel). 2020 Dec 3;9(12):861. doi: 10.3390/antibiotics9120861 (PMC7761637; doi:10.3390/antibiotics9120861)
Supplement: Supplementary file 1 [file antibiotics-09-00861-s001.pdf]

Supplementary material

Article

Comprehensive statistical evaluation of Etest®, UMIC®, MicroScan and disc diffusion versus standard broth microdilution: workflow for an accurate detection of colistin-resistant and *mcr*-positive *E. coli*

Isidro García-Meniño [1,2], Pilar Lumbreras [3,4], Pablo Valledor [5], Dafne Díaz-Jiménez [1,2], Luz Lestón [1,2], Javier Fernández [3,4,5,*,†] and Azucena Mora [1,2,*,†]

1 Laboratorio de Referencia de *Escherichia coli* (LREC), Departamento de Microbioloxía e Parasitoloxía, Facultade de Veterinaria, Universidade de Santiago de Compostela (USC), 27002 Lugo, Spain; isidro.garcia@usc.es (I.G.-M.); dafne.diaz@usc.es (D.D.-J.); luz.cambeiro@usc.es (L.L.)

2 Instituto de Investigación Sanitaria de Santiago de Compostela (IDIS), 15706 Santiago, Spain

3 Servicio de Microbiología, Hospital Universitario Central de Asturias, 33011 Oviedo, Spain; UO231178@uniovi.es

4 Grupo de Microbiología Traslacional, Instituto de Investigación Sanitaria del Principado de Asturias (ISPA), 33011 Oviedo, Spain

5 Research & Innovation, Artificial Intelligence and Statistical Department, iAST™, 33007 Oviedo, Spain; pablo.valledor@iast.ai (P.V.)

* Correspondence: javifdom@gmail.com (J.F.); azucena.mora@usc.es (A.M.)

† These authors contributed equally to this paper.

Table S1. Results of **c**olistin susceptibility for 75 *mcr*-positive *E. coli* isolates using BMD, UMIC, MicroScan, gradient diffusion strip (Etest®) and disc diffusion.

ID	[1]Origin	*mcr* gene	[2]BMD (MICs)	[2]UMIC® (MICs)	[2]MicroScan (MICs)	[2]ETEST (MICs)	[2]Disc diffusion (≤11 mm)	[3]Disc diffusion (≤13 mm mm)	[4]Colistin point mutation
1-COL	Porcine diarrhea	*mcr-1*	4	8	>4	6	13	13	None
2-COL	Porcine diarrhea	*mcr-1*	4	8	>4	6	11	11	None
3-COL	Porcine diarrhea	*mcr-1*	4	4	>4	4	11	11	None
4-COL	Porcine diarrhea	*mcr-1*	8	8	>4	8	9	9	*pmr*B V161G
5-COL	Porcine diarrhea	*mcr-1*	8	16	>4	4	9	9	None
6-COL	Porcine diarrhea	*mcr-1*	4	4	>4	4	11	11	None
8-COL	Porcine diarrhea	*mcr-1*	4	8	>4	6	11	11	None
9-COL	Porcine diarrhea	*mcr-1*	4	8	>4	8	11	11	*pmr*A S39I
10-COL	Porcine diarrhea	*mcr-1*	2	4	>4	3	13	13	*pmr*A S39I
13-COL	Porcine diarrhea	*mcr-1*	8	8	>4	12	9	9	None
14-COL	Porcine diarrhea	*mcr-1*	4	4	>4	4	12	12	None
15-COL	Porcine diarrhea	*mcr-1*	4	4	>4	4	12	12	None
16-COL	Porcine diarrhea	*mcr-1*	4	8	>4	8	11	11	*pmr*B V161G
17-COL	Porcine diarrhea	*mcr-1*	2	4	>4	6	12	12	None
44-COL	Porcine diarrhea	*mcr-1*	8	8	>4	8	10	10	None
45-COL	Porcine diarrhea	*mcr-1*	4	4	>4	6	11	11	None
46-COL	Porcine diarrhea	*mcr-1*	8	8	>4	8	10	10	None
67-COL	Porcine diarrhea	*mcr-1*	2	4	>4	4	13	13	NP
74-COL	Porcine diarrhea	*mcr-1*	4	4	>4	4	11	11	NP
76-COL	Porcine diarrhea	*mcr-1*	8	8	>4	12	9	9	NP
77-COL	Porcine diarrhea	*mcr-1*	4	4	>4	4	11	11	NP
78-COL	Porcine diarrhea	*mcr-1*	2	4	>4	4	11	11	NP
33a-COL	Porcine diarrhea (ST131)	*mcr-1*	8	16	>4	12	8	8	None
34a-COL	Porcine diarrhea (ST131)	*mcr-1*	4	8	>4	6	11	11	None
35a-COL	Porcine diarrhea (ST131)	*mcr-1*	4	4	>4	6	11	11	NP
36-COL	Porcine diarrhea (ST131)	*mcr-1*	4	4	>4	4	11	11	NP
37-COL	Porcine diarrhea (ST131)	*mcr-1*	4	4	>4	6	11	11	NP
38-COL	Porcine diarrhea (ST131)	*mcr-1*	4	4	>4	6	12	12	NP
39-COL	Pork meat (ST131)	*mcr-1*	4	8	>4	6	11	11	None
40-COL	Pork meat (ST131)	*mcr-1*	4	8	>4	6	11	11	None
41-COL	Pork meat (ST131)	*mcr-1*	8	8	>4	8	10	10	None
42-COL	Pork meat (ST131)	*mcr-1*	4	8	>4	4	11	11	None
43b-COL	Pork meat (ST131)	*mcr-1*	4	4	>4	6	11	11	NP
48-COL	Chicken meat	*mcr-1*	4	8	>4	6	10	10	NP
49-COL	Chicken meat	*mcr-1*	4	8	>4	4	11	11	NP
50-COL	Chicken meat	*mcr-1*	4	4	>4	3	12	12	NP
51-COL	Chicken meat	*mcr-1*	4	8	>4	8	10	10	NP
53-COL	Chicken meat	*mcr-1*	4	4	>4	6	11	11	NP
54-COL	Chicken meat	*mcr-1*	4	4	>4	6	11	11	NP
55-COL	Chicken meat	*mcr-1*	2	4	>4	4	13	13	NP

56-COL	Chicken meat	*mcr-1*	1	4	>4	3	12	12	NP
57-COL	Beef meat	*mcr-1*	8	4	>4	6	12	12	NP
58-COL	Wildlife feces	*mcr-1*	4	4	>4	4	11	11	NP
85-COL	Porcine diarrhea	*mcr-2*	4	4	>4	4	12	12	NP
18-COL	Porcine diarrhea	*mcr-4.1*	4	4	>4	6	12	12	None
19-COL	Porcine diarrhea	*mcr-4.2*	8	8	>4	16	10	10	None
20-COL	Porcine diarrhea	*mcr-4.2*	2	4	>4	6	10	10	*pmr*B V161G
21-COL	Porcine diarrhea	*mcr-4.2*	2	4	>4	4	12	12	None
22-COL	Porcine diarrhea	*mcr-4.2*	2	4	>4	8	11	11	None
23-COL	Porcine diarrhea	*mcr-4.2*	2	4	>4	4	11	11	*pmr*B V161G
24-COL	Porcine diarrhea	*mcr-4.2*	4	4	>4	6	13	13	*pmr*B V161G
25-COL	Porcine diarrhea	*mcr-4.2*	4	4	>4	6	11	11	None
26-COL	Porcine diarrhea	*mcr-4.2*	4	4	>4	4	11	11	*pmr*B V161G
27-COL	Porcine diarrhea	*mcr-4.2*	4	8	>4	8	9	9	*pmr*B V161G
28-COL	Porcine diarrhea	*mcr-4.2*	2	4	>4	4	13	13	None
29-COL	Porcine diarrhea	*mcr-4.2*	8	8	>4	6	10	10	*pmr*B V161G
30-COL	Porcine diarrhea	*mcr-4.5*	4	8	>4	8	10	10	*pmr*B V161G
31-COL	Porcine diarrhea	*mcr-4.5*	4	4	>4	6	11	11	*pmr*B V161G
79-COL	Porcine diarrhea	*mcr-4.5*	4	4	>4	6	11	11	NP
80-COL	Porcine diarrhea	*mcr-4.2*	2	4	>4	4	12	12	NP
81-COL	Porcine diarrhea	*mcr-4.5*	4	4	>4	4	11	11	NP
59-COL	Porcine diarrhea	*mcr-4.2*	4	4	>4	6	11	11	NP
60-COL	Porcine diarrhea	*mcr-4.5*	4	8	>4	4	12	12	NP
61-COL	Porcine diarrhea	*mcr-4.5*	4	8	>4	6	11	11	NP
62-COL	Porcine diarrhea	*mcr-4.2*	8	8	>4	8	9	9	NP
63-COL	Porcine diarrhea	*mcr-4.2*	4	4	>4	6	10	10	NP
65-COL	Porcine diarrhea	*mcr-4.2*	4	4	>4	4	12	12	NP
70-COL	Porcine diarrhea	*mcr-4.2*	4	4	>4	4	11	11	NP
32-COL	Porcine diarrhea	*mcr-5.1*	2	4	>4	3	12	12	*pmr*B V161G
33b-COL	Porcine diarrhea	*mcr-5.1*	4	4	>4	6	11	11	*pmr*B V161G
34-COL	Porcine diarrhea	*mcr-1 , mcr-4*	4	4	>4	8	9	9	*pmr*B V161G
35-COL	Porcine diarrhea	*mcr-1, mcr-4*	4	8	>4	8	11	11	*pmr*B V161G
84-COL	Porcine diarrhea	*mcr-1, mcr-4*	8	8	>4	6	11	11	NP
87-COL	Porcine diarrhea	*mcr-4, mcr-5*	4	2	>4	3	13	13	NP
71-COL	Porcine diarrhea	*mcr-4, mcr-5*	4	4	>4	4	11	11	NP

[1] Origin of isolation: colibacillosis diagnosis (porcine diarrhea); meat sampled at retails (chicken, pork and beef meat); fox (wildlife feces). *mcr*-positive isolates belonging to the ST131 lineage of *E. coli* are indicated in parentheses.[2] Susceptible values when applying the colistin clinical cut-off s established by EUCAST 2020 are highlighted in grey: MIC cut-off >2 mg/L; Diameter inhibition cut-off ≤ 11 (mm).[3] All 75 *mcr*-positive isolates would be determined as resistant for the disc diffusion assay when applying the diameter inhibition cut-off ≤13 (mm) proposed here. [4]Colistin point mutation analysed in silico. (García-Meniño et al. 2019. Front Microbiol 10). NP: not performed.

Table S2. Results of **c**olistin susceptibility for 75 *mcr*-negative *E. coli* isolates using BMD, UMIC, MicroScan, gradient diffusion strip (Etest®) and disc diffusion.

ID	[1]Origin	[2]BMD (MICs)	[2]UMIC® (MICs)	[2]MicroScan (MICs)	[2]ETEST (MICs)	[2]Disc diffusion (≤11 mm)	[3]Disc diffusion (≤13 mm)
S1	Porcine diarrhea	0.125	0.25	<2	0.19	16	16
S2	Porcine diarrhea	0.125	0.25	<2	0.5	14	14
S3	Porcine diarrhea	0.125	0.25	<2	0.25	14	14
S4	Porcine diarrhea	0.125	0.25	<2	0.19	14	14
S5	Porcine diarrhea	0.125	0.25	<2	0.19	14	14
S6	Porcine diarrhea	0.25	0.25	<2	0.25	15	15
S7	Porcine diarrhea	0.125	0.25	<2	0.19	13	13
S8	Porcine diarrhea	0.125	0.25	<2	0.25	14	14
S9	Porcine diarrhea	0.25	0.5	<2	0.19	15	15
S10	Porcine diarrhea	0.125	0.25	<2	0.38	14	14
S11	Porcine diarrhea	0.125	0.25	<2	0.25	16	16
S12	Porcine diarrhea	0.125	0.25	<2	0.19	15	15
S13	Porcine diarrhea	0.125	0.25	<2	0.125	15	15
S14	Porcine diarrhea	0.125	0.25	<2	0.25	15	15
S15	Porcine diarrhea	0.125	0.25	<2	0.25	14	14
S16	Porcine diarrhea	0.125	0.25	<2	0.125	14	14
S17	Porcine diarrhea	0.125	0.25	<2	0.19	15	15
S18	Porcine diarrhea	0.25	0.25	<2	0.19	17	17
S19	Porcine diarrhea	0.125	0.25	<2	0.125	17	17
S20	Porcine diarrhea	0.125	0.25	<2	0.19	18	18
S21	Porcine diarrhea	0.125	0.25	<2	0.094	18	18
S22	Porcine diarrhea	0.125	0.25	<2	0.094	16	16
S23	Porcine diarrhea	0.125	0.125	<2	0.094	17	17
S24	Porcine diarrhea	0.125	0.25	<2	0.047	18	18
S25	Porcine diarrhea	0.125	0.25	<2	0.19	17	17
S26	Porcine diarrhea	0.25	0.25	<2	0.094	16	16
S27	Porcine diarrhea	0.125	0.25	<2	0.125	15	15
S28	Porcine diarrhea	0.125	0.5	<2	0.125	16	16
S29	Porcine diarrhea	0.125	0.25	<2	0.125	15	15
S30	Porcine diarrhea	0.125	0.25	<2	0.125	15	15
S31	Porcine diarrhea	0.125	0.25	<2	0.125	15	15
S32	Porcine diarrhea	0.125	0.25	<2	0.125	16	16
S33	Porcine diarrhea	0.125	0.25	<2	0.25	15	15
S34	Porcine diarrhea	0.125	0.25	<2	0.125	15	15
S35	Porcine diarrhea	0.125	0.25	<2	0.125	16	16
S36	Porcine diarrhea (ST131)	0.125	0.25	<2	0.094	16	16
S37	Porcine diarrhea (ST131)	0.125	0.25	<2	0.25	16	16
S38	Porcine diarrhea (ST131)	0.125	0.25	<2	0.125	15	15
S39	Porcine diarrhea (ST131)	0.125	0.25	<2	0.125	17	17

S40	Porcine diarrhea (ST131)	0.125	0.5	<2	0.25	20	20
S41	Porcine diarrhea (ST131)	0.125	0.25	<2	0.125	17	17
S42	Porcine diarrhea (ST131)	0.125	0.25	<2	0.125	15	15
S43	Porcine diarrhea (ST131)	0.125	0.25	<2	0.125	16	16
S44	Porcine diarrhea (ST131)	0.25	0.5	<2	0.38	16	16
S45	Porcine diarrhea (ST131)	0.125	0.25	<2	0.125	16	16
S46	Porcine diarrhea (ST131)	0.125	0.25	<2	0.064	16	16
S47	Pork meat (ST131)	0.125	0.25	<2	0.125	15	15
S48	Pork meat (ST131)	0.125	0.25	<2	0.125	15	15
S49	Pork meat (ST131)	0.125	0.5	<2	0.19	16	16
S50	Pork meat (ST131)	0.25	0.5	<2	0.125	16	16
S51	Pork meat (ST131)	0.25	0.25	<2	0.125	16	16
S52	Pork meat (ST131)	0.125	0.25	<2	0.047	16	16
S53	Pork meat (ST131)	0.125	0.5	<2	0.125	16	16
S54	Pork meat (ST131)	0.25	0.5	<2	0.125	15	15
S55	Pork meat (ST131)	0.25	0.25	<2	0.094	16	16
S56	Pork meat (ST131)	0.25	0.5	<2	0.25	15	15
S57	Pork meat (ST131)	0.125	0.25	<2	0.125	17	17
S58	Pork meat (ST131)	0.125	0.5	<2	0.125	16	16
S59	Pork meat (ST131)	0.5	0.5	<2	0.125	16	16
S60	Beef meat	0.125	0.5	<2	0.75	15	15
S61	Beef meat	0.125	0.125	<2	0.125	16	16
S62	Beef meat	0.125	0.25	<2	0.25	15	15
S63	Chicken meat	0.125	0.25	<2	0.125	15	15
S64	Chicken meat	0.125	0.25	<2	0.19	14	14
S65	Chicken meat	0.125	0.25	<2	0.125	14	14
S66	Chicken meat	0.125	0.25	<2	0.125	15	15
S67	Chicken meat	0.125	0.25	<2	0.125	15	15
S68	Chicken meat	0.125	0.25	<2	0.38	15	15
S69	Chicken meat	0.125	0.25	<2	0.125	15	15
S70	Chicken meat	0.125	0.25	4	0.125	15	15
S71	Chicken meat	0.125	0.25	<2	0.125	15	15
S72	Chicken meat	0.125	0.25	<2	0.19	14	14
S73	Chicken meat	0.125	0.25	<2	0.094	15	15
S74	Chicken meat	0.125	0.5	<2	0.125	16	16
S76	Chicken meat	1	0.5	<2	0.094	16	16

[1] Origin of isolation: colibacillosis diagnosis (porcine diarrhea); meat sampled at retails (chicken, pork and beef meat). *mcr*-negative isolates belonging to the ST131 lineage of *E. coli* are indicated in parentheses.[2] Non-susceptible values when applying the colistin clinical cut-off s established by EUCAST 2020 are highlighted in grey: MIC cut-off > 2 mg/L; Diameter inhibition cut-off ≤ 11 (mm).[3] Non-susceptible values when applying the diameter inhibition cut-off ≤ 13 (mm) proposed here are highlighted in grey. This collection was not analysed for colistin point mutation.
